# Mixing Rules for an Exact Determination of the Dielectric Properties of Engine Soot Using the Microwave Cavity Perturbation Method and Its Application in Gasoline Particulate Filters

**DOI:** 10.3390/s22093311

**Published:** 2022-04-26

**Authors:** Stefanie Walter, Peter Schwanzer, Carsten Steiner, Gunter Hagen, Hans-Peter Rabl, Markus Dietrich, Ralf Moos

**Affiliations:** 1 Bayreuth Engine Research Center (BERC), Department of Functional Materials, University of Bayreuth, 95447 Bayreuth, Germany; stefanie.walter@uni-bayreuth.de (S.W.); carsten.steiner@uni-bayreuth.de (C.S.); gunter.hagen@uni-bayreuth.de (G.H.); 2 Ostbayerische Technische Hochschule Regensburg, 93053 Regensburg, Germany; peter.schwanzer@oth-regensburg.de (P.S.); hans-peter.rabl@oth-regensburg.de (H.-P.R.); 3 Vitesco Technologies, 93055 Regensburg, Germany; markus.dietrich@vitesco.com

**Keywords:** gasoline particulate filter (GPF), radio-frequency (RF), soot mass determination, finite element method (FEM), microwave cavity perturbation, dielectric properties, mixing rule

## Abstract

In recent years, particulate filters have become mandatory in almost all gasoline-powered vehicles to comply with emission standards regarding particulate number. In contrast to diesel applications, monitoring gasoline particulate filters (GPFs) by differential pressure sensors is challenging due to lower soot masses to be deposited in the GPFs. A different approach to determine the soot loading of GPFs is a radio frequency-based sensor (RF sensor). To facilitate sensor development, in previous work, a simulation model was created to determine the RF signal at arbitrary engine operating points. To ensure accuracy, the exact dielectric properties of the soot need to be known. This work has shown how small samples of soot-loaded filter are sufficient to determine the dielectric properties of soot itself using the microwave cavity perturbation method. For this purpose, mixing rules were determined through simulation and measurement, allowing the air and substrate fraction of the sample to be considered. Due to the different geometry of filter substrates compared to crushed soot samples, a different mixing rule had to be derived to calculate the effective filter properties required for the simulation model. The accuracy of the determined mixing rules and the underlying simulation model could be verified by comparative measurements on an engine test bench.

## 1. Introduction

In recent years, gasoline engines have gained attention within the public discourse regarding automotive pollutant emissions and, in particular, particulate emissions. Although they emit a significantly lower soot mass compared to diesel engines, the number of emitted particles is not negligible due to their smaller size. Studies also show that the number of small particles and not only their mass affects human health negatively [1,2,3]. Especially direct injection systems, which reduce fuel consumption compared to intake manifold injection, lead to increased soot particulate emissions due to the inhomogeneous mixture of fuel and intake air in the combustion chamber [4]. Whereas exhaust emission standards limiting only particulate mass (PM) could be met by engine-based measures, this is no longer sufficient for a large proportion of vehicles due to the limitation of the particle number (PN) within the Euro 6b standard. In particular, since the introduction of Real-Driving-Emissions (RDE), these limits can only be met by installing gasoline particulate filters (GPFs) [5,6].

To ensure fault-free operation, monitoring the soot loading of particulate filters is advantageous. The state-of-the-art for diesel particulate filters (DPFs) is a loading model based on the differential backpressure (Δ*p*), which drops along the filter [7]. Such a sensor system can also be used for gasoline particulate filters, but due to different engine operation and differences in the filter system itself, an accurate determination of the stored soot mass is not possible under all circumstances [8,9,10,11,12,13]. Due to smaller particle sizes in gasoline applications, less soot mass accumulates in the GPF and therefore a lower backpressure increase has to be detected. In addition, a higher exhaust gas mass flow leads to a generally higher backpressure. As a consequence, a significantly lower relative signal change has to be measured compared to diesel applications [13]. Furthermore, unintended regenerations can occur more frequently due to the higher reactivity of gasoline soot at simultaneously higher exhaust gas temperatures [14,15]. This can lead to partial regenerations and, due to the resulting changed gas flow through the filter walls, to a lower differential pressure than expected [9,10,16,17].

For diesel applications, a radio frequency-based sensor (RF sensor) has been developed as an alternative to the conventional load detection [18,19,20,21,22,23]. The RF sensor can be used to determine the amount of deposited soot directly via the dielectric properties of the filter. In addition, the strongly divergent dielectric properties of ash allow it to be distinguished from soot [21]. Despite the numerous differences between diesel and gasoline applications, the functionality of the RF sensor regarding GPFs has already been demonstrated [8,17,24,25]. Furthermore, for catalytically coated GPFs (cGPFs), monitoring of the catalyst state is feasible. For instance, in three-way catalysts (TWC), the oxidation degree of ceria is directly linked with the RF sensor signal [26]. This correlation could also be shown for cGPFs, at least for soot-free conditions [25].

However, a direct application of the RF sensor without prior calibration to engine and exhaust system is not possible. The dielectric properties of soot, and thus the sensitivity of the RF sensor, are influenced by parameters such as the engine load or the filter temperature [8]. To correct these cross-sensitivities, extensive engine test bench measurements can be necessary. The required effort can be reduced by the simulation model developed in a previous work, which allows the replication of different operating conditions and calculates their effect on the RF as well as the differential pressure signal [10]. For an accurate signal computation, the dielectric properties of the accumulated soot, respective to its influence on the effective filter properties, must be known.

For that purpose, the soot properties can be determined by measuring soot-loaded filter samples using the microwave cavity perturbation (MCP) method [27,28]. In order to deduce accurate material properties, mixing rules have to be applied [27]. They are necessary because soot cannot be measured directly, rather, only the effective properties of the mixture together with filter substrate and air. However, the applicable mixing rule depends not only on the sample geometry, but also on the mixture components themselves [29]. In this paper, mixing rules for particulate filters are determined. Therefore, first, the influence of air content on the effective dielectric properties is simulated for a different mixture with filter substrate. This is done not only for the geometry as it is typically measured in the MCP setup (i.e., a filter crushed to powder) but also for the monolith structure of an intact GPF. In order to deduce the properties of the soot itself from those of the soot-loaded substrate, a second mixing rule must be applied. This is determined by measuring differently loaded filters in an MCP resonator. These mixing rules can then be used to deduce the effective dielectric properties of a filter with any soot loading using a small filter sample. To validate the determined mixing rules, data obtained on an engine test bench were compared with calculations of the simulation model presented in [10] using these mixing rules.

## 2. Fundamentals of the MCP and Their Application to Soot-Loaded Particulate Filters

### 2.1. Determination of Dielectric Properties Using the MCP

A direct measurement of the dielectric properties of GPFs mounted in the exhaust is not possible due to multiple unknown influencing factors, such as the exact temperature distribution across the filter and the impossibility of calibration with an empty filter canning. To, nevertheless, determine the exact properties of soot and filter substrate, the microwave cavity perturbation (MCP) method is suitable. For this, a material sample is brought into a resonant cavity. Its dielectric properties, which can be described by the complex relative permittivity $\varepsilon_{r}$, thereby influence the excited resonant mode [30]. $\varepsilon_{r}$ is composed of the relative dielectric constant $\varepsilon_{r}^{\prime }$ and the dielectric losses $\varepsilon_{r}^{\prime \prime }$, which are caused not only by losses due to sample polarization $\varepsilon_{r,\mathrm{pol}}^{\prime \prime }$ but also by conductivity losses σ (Equation (1)). The latter also depends on the vacuum permittivity $\varepsilon_{0\;}$ and the angular frequency of the excited electromagnetic field ω=2πf.

(1) $$\varepsilon_{r}=\varepsilon_{r}^{\prime }-{j\varepsilon }_{r}^{\prime \prime }=\varepsilon_{r}^{\prime }-j\left(\varepsilon_{r,\mathrm{pol}}^{\prime \prime }+\frac{\sigma }{\varepsilon_{0\;}\omega }\right)$$

When the sample is placed in the middle of a cylindrical cavity, based on Maxwell’s equations, the resulting shift of resonant frequency Δf relative to the frequency without a sample $f_{0}$ can be associated to $\varepsilon_{r}^{\prime }$ (Equation (2)), and the changing inverse quality factor ${\Delta Q}^{-1}$ can be related to the dielectric losses $\varepsilon_{r}^{\prime \prime }$ (Equation (3)) [30,31,32]. In addition to the sample volume $V_{s}$, an effective resonant volume $V_{\mathrm{eff}}$ has to be considered, which can be determined by the field distribution of the resonant mode [32].

(2) $$\frac{\Delta f}{f_{0}}=\left(\varepsilon_{r}^{\prime }-1\right)\;\frac{V_{s}}{{2V}_{\mathrm{eff}}}$$

(3) $$\Delta \left(Q^{-1}\right)={\;\varepsilon }_{r}^{\prime \prime }\;\frac{V_{s}}{V_{\mathrm{eff}}}$$

One requirement for the validity of these equations is that the electromagnetic field is not affected by inserting the sample. In reality, this cannot be assumed, especially for samples with high permittivity or high dielectric losses. Nevertheless, it is possible to address these deviations from the simplified theory. In particular, the following three issues have to be considered for an accurate measurement of the sample [27]:

- Changes in the electromagnetic field due to depolarization inside the sample.
- Deviation of the field distribution in the cavity due to its non-ideal cylindrical shape.
- Necessity to apply mixing rules for porous samples or samples with multiple species.

The first two aspects are independent of the dielectric properties of the sample and can be addressed even if only the geometry of the sample and resonant cavity is known [28,33,34]. Mixing rules, on the other hand, also depend on the properties of the sample and will be discussed in more detail in Section 2.2 and Section 2.3 [34].

**Figure 1:**
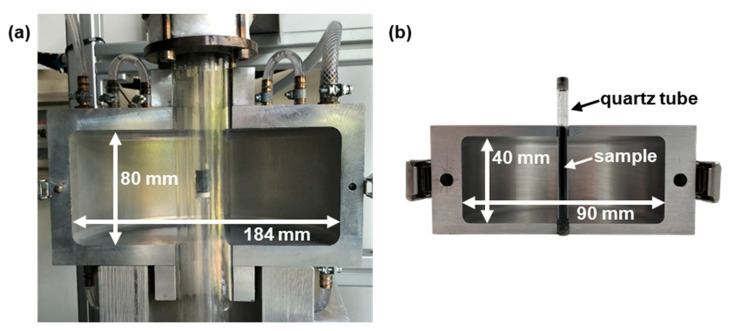
Images of the resonators used in this work. (**a**) Resonator with possibility to heat the sample; described in more detail in [28]. (**b**) Non-heatable resonator with smaller cavity compared to (**a**).

In this work, two resonators of different sizes were used. With the resonator shown in Figure 1a, samples can be heated up to 600 °C and simultaneously exposed to different gas atmospheres. A detailed description of the resonator setup can be found in [28]. It is used to determine temperature-dependent dielectric properties of soot. Therefore, after measuring a soot-loaded filter, the soot is removed in an oxidizing atmosphere at 600 °C and the then soot-free substrate is measured again. After considering the air content in the sample, the difference between the two measurements can be used to deduce the soot properties by applying the mixing rule to be determined in Section 3.2.

To examine this mixing rule, a smaller, simpler resonator is used (Figure 1b), which was already utilized in [27] to determine the mixing rule between ceria and air. Its cylindrical cavity is 90 mm in diameter and 40 mm high. The sample tube is made of quartz glass to avoid additional dielectric losses as much as possible and has an inner diameter—which corresponds to the diameter of the sample—of 3 mm. As with the larger resonator, the evaluated resonance corresponds to the TM_010_ mode and occurs at a frequency of approx. 2.48 GHz. Compared to the larger resonator, this one is not capable of adjusting the sample temperature or gas atmosphere, allowing a simpler design of the sample tube. Hence, the cavity has smaller openings, which results in smaller deviations of the electromagnetic field in comparison to a perfectly cylindrical resonator. Additionally, the larger resonator can only be filled with small sample heights to allow an unhindered gas flow to set defined gas atmospheres. To nevertheless position the material sample centrically in the cavity, a porous quartz glass frit is mounted inside the sample tube. In contrast, in the smaller resonator, the sample can completely traverse the cavity, resulting in no depolarization effects due to the field distribution of the observed resonant mode. Thus, using the smaller resonator, the simplified MCP can be applied without the adjustments described in [27], and applicable mixing rules can be determined with less possible interferences. Due to the smaller sample diameter as a result of the generally smaller resonator dimensions, no more sample volume than for the measurements in the large resonator is required, despite the completely filled quartz tube.

### 2.2. Influence of Mixing Rules on the Material Property Determination

Mixing rules describe the relationship of the effective complex permittivity related to the dielectric properties of the mixture components depending on their volume faction. The applicability of a mixing rule depends strongly on the material itself [35,36,37,38,39,40,41,42]. Thus, a variety of different mixing rules is reported in literature [43,44,45,46,47,48,49]. One reason for those strongly different rules lies in the structure of the individual particles. The electromagnetic field in each particle is depolarized depending on its shape. In average, the resulting depolarization across the entire mixture then leads to the measurable effective permittivity [29]. Therefore, depending on the proportion of the mixing components, polarization effects may influence each other, leading to different mixing rules [34,50].

**Figure 2:**
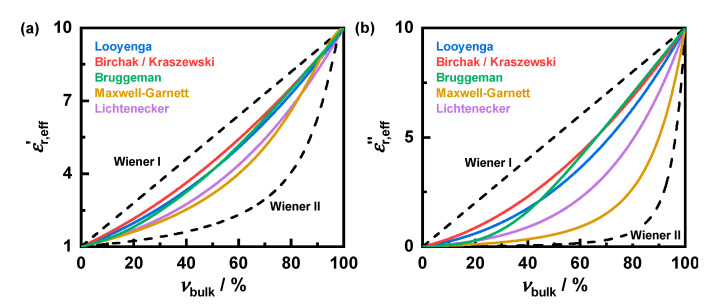
Example for application of different mixing rules on the effective permittivity $\varepsilon_{r,\mathrm{eff}}^{\prime }$ (**a**) and the dielectric losses $\varepsilon_{r,\mathrm{eff}}^{\prime \prime }$ (**b**) depending on the bulk fraction $\nu_{\mathrm{bulk}}$ for mixtures of air ($\varepsilon_{r,\mathrm{air}}^{\prime }$ = 1; $\varepsilon_{r,\mathrm{air}}^{\prime \prime }$ = 0) and lossy material (exemplarily values: $\varepsilon_{r,\mathrm{bulk}}^{\prime }$ = 10; $\varepsilon_{r,\mathrm{bulk}}^{\prime \prime }$ = 10). Mixing rules according to Looyenga [44], Birchak [45], Bruggeman [46], Maxwell-Garnett [47], Lichtenecker [48] and Wiener for a series (Wiener I) as well as for a parallel circuit (Wiener II) of the mixture components [49].

Usually, mixing rules refer only to the real part of the permittivity. For the Maxwell–Garnett rule, lossy materials are discussed theoretically in [29]. Considerations about other mixing rules or measurements to validate these regarding dielectric losses are barely found in the literature. By considering the complex permittivity (cf. Equation (1)) for the mixing rules, the theoretical dependencies of the dielectric losses on the material fraction can be determined (Figure 2). Although a simple analytical function cannot be found for most mixing rules, a numerical determination of the effective dielectric losses $\varepsilon_{r,\mathrm{eff}}^{\prime \prime }$ is possible. The exact behavior of the effective properties depends on both the losses of the mixture components and their permittivity. The shape of the thereby resulting effective dielectric losses differs significantly from that of the effective permittivity. Furthermore, losses do not have to obey the same mixing rule as the permittivity. This is only the case if both are caused by the same material effect. For soot-loaded filters, this may not be the case, since their dielectric losses are mainly influenced by the soot conductivity, whereas their permittivity is affected by both soot and filter substrate. Therefore, for particulate filters, it is not feasible to derive the mixing rule regarding losses from that for the permittivity.

Only the loss-based mixing rules will be examined in this work, since, in particulate filter applications, the RF signal depends primarily on the effective filter losses. To simplify the examination of the mixing rules, they will be evaluated using an exponential approach, according to Equation (4).

(4) $${\left(\varepsilon_{r,\mathrm{eff}}^{\prime \prime }\right)}^{k}=\overset{n}{\underset{i}{\sum }}\nu_{i}{\left(\varepsilon_{r,i}^{\prime \prime }\right)}^{k}$$

Thereby, the losses of the mixing components $\varepsilon_{r,i}^{\prime \prime }$ to the power of *k* contribute to the effective permittivity $\varepsilon_{r,\mathrm{eff}}^{\prime \prime }$ according their volume fraction $\nu_{i}$ [35]. This power law is also used in various mixing rules, such as by Looyenga [44] with an exponent of 1/3 or by Birchak [45] with 1/2.

A theoretical evaluation of which mixing rule respective to the exponent has to be applied for GPFs is hardly possible due to the random arrangement of the mixing components, especially in the porous structure of substrate walls. However, it can be obtained by measuring the effective properties at different compositions of the mixture components. Equivalent to this method, mixing rules can also be determined simulatively by finite element analysis (FEA). However, especially for finely distributed mixture components, as is the case for soot particles deposited in a filter, a calculation of the electromagnetic field, which is responsible for the measurable effective permittivity, would only be possible with high computational efforts.

### 2.3. Possible Mixing Rules for Soot-Loaded Filter

For particulate filters, a mixture of three different components—soot, filter substrate and air—has to be accounted, whereby the application of a common mixing rule seems impractical due to the different distribution of the components. Therefore, two independent mixing rules should be used to describe the effective dielectric properties. One involves the interaction between the soot-loaded filter substrate and air. The other examines the mixture of soot itself with substrate, which in this work is cordierite, as is typically used in GPF applications.

Literature mainly discusses mixing rules that address the air content in relation to a bulk medium [39,40,41,42,43]. Mixtures of two components, such as polymers filled with carbon black [37] or moisture in a solid material [38,45], are also investigated. Regarding the dielectric properties of GPFs, however, descriptions of the mixing behavior between filter substrate and soot deposited on it, especially regarding their losses, cannot be found. Therefore, the interaction between them will be investigated in Section 3.2. On the contrary, regarding the second required mixing rule, there are studies concerning the influence of air on cordierite, although not for a soot-loaded case. In [40], Wiener’s rule for a series circuit was found as suitable regarding the effective permittivity. However, only low porosities of up to 5% were examined. Whether this mixing rule also applies at higher air contents, as is the case for GPF applications, cannot be verified. Furthermore, deviating from this, the data from [39] find agreements with the Looyenga mixing rule. However, only samples with porosities around 20% respective to 80% were examined, and no investigations were conducted regarding dielectric losses. Due to this ambiguous literature, the mixing rule will be determined in Section 3.1.

## 3. Determination of the Mixing Rules for Soot-Loaded Particulate Filters

### 3.1. Soot-Loaded Filter-Air Mixing Rule

To obtain the dielectric soot properties, first, the effective parameters of soot-loaded filter substrate must be derived. Therefore, the influence of air must be eliminated using a mixing rule. To identify a valid mixing rule, measurements with the smaller resonator (Figure 1b) were attempted. Although it was possible to vary the air content by compressing the sample, this was only possible, similar to the studies discussed in Section 2.3, within a small range. A variation in the air content to a greater extent would have been possible by, e.g., milling. However, this would change the macroscopic structure of the porous substrate and would, consequently, influence the applicable mixing rule. Thus, only limited evidence could be drawn from these measurements. As an alternative, the mixing rule will be determined by simulation using the FEA-software COMSOL Multiphysics^®^ 5.6.

To ensure comparability of the electromagnetic field distribution, a cylindrical resonator with the same dimensions as the larger resonator (Figure 1a, described in more detail in [28]) is simulated. For simplicity, the quartz glass structure for sample heating and adjustment of the gas atmosphere is not included in the model. Moreover, coupling elements (antennas) to excite and receive electromagnetic waves are not modeled. Instead, the resonant modes are determined by modal analysis. As a result, the simulated resonator is equivalent to a simple cylindrical cavity, whereby only the sample influences the resonant properties according to the simplified MCP theory (cf. Equations (2) and (3)). Thus, the mixing rule can be analyzed independently of disturbing influences. In addition, the resonator can be considered as rotationally symmetric since the resonant mode to be examined is, as in the real resonator, the TM_010_ mode, which has no azimuthal dependence of the electromagnetic field. This allows for calculation of the resonant parameters in a two-dimensional model. Similar to the real measurement setup, the cylindrical sample (10 mm in diameter) is located in the center of the resonator, but passes completely through the resonator. This prevents depolarization effects, as described in [27]. Despite the two-dimensional approach, an exact replication of the particles is not possible without great computational effort. Therefore, the substrate–air mixture of the sample will be modeled in simplified form. Since, in the resonator setup only coarsely crushed filter substrates are measured to keep the sample as similar as possible to an intact particulate filter, the sample bulk in the simulation model is assumed as multiple cylindrical layers of filter substrate stacked upon each other separated by air. The substrate fraction $\nu_{\mathrm{bulk}}$ is adjusted by the height of the layers. In addition, the influence of GPFs with different degrees of soot loading is accounted by varying the substrate conductivity, which is the only source of dielectric losses ($\varepsilon_{r,\mathrm{pol}}^{\prime \prime }$ << $\frac{\sigma }{\varepsilon_{0\;}\omega }$). The parameter values that varied in the simulation are listed in Table 1. The substrate permittivity, meanwhile, is not varied and has a value of $\varepsilon_{r}^{\prime }$ = 1.5, corresponding to the effective properties of soot-loaded GPF samples measured in this work by the MCP method. 

**Figure 3:**
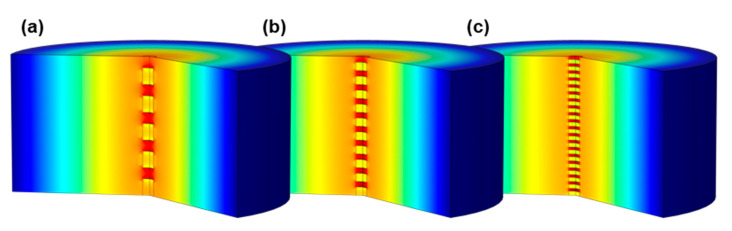
Simulated electric field (red: high field strength; blue: low field strength) of the TM_010_ resonant mode in a rotationally symmetric resonator setup with a centered sample split into several layers ($\nu_{\mathrm{bulk}}$ = 60%; $\sigma_{\mathrm{bulk}}$ = 40 mS/m): (**a**) 5 layers, (**b**) 10 layers and (**c**) 20 layers.

The resulting electric field distribution for different numbers of layers is shown in Figure 3. A decrease in field strength inside the substrate layers combined with an increase within the intermediate air can be observed. This polarization causes lower effective dielectric properties and becomes stronger with an increasing number of layers. How exactly this affects the resulting mixing rule can be determined via the dependence of the resonant parameters on the substrate volume fraction $\nu_{\mathrm{bulk}}$.

For a correct simulation of the RF sensor signal during GPF monitoring, the effective properties of an intact filter structure must also be derived. Based on the properties of soot-loaded substrates, the air content, which differs from the sample measured in the resonator setup, must be considered. The applicable mixing rule may differ due to the deviating sample geometry and a different excited resonant mode. It will be determined by simulating the parallel channels of a monolithic filter structure. In contrast to the resonator setup, the model therefore has to be three-dimensional. To reduce the computational effort nevertheless, simplifications compared to a real filter are made. Thus, the plugs of the alternately closed channels or the smooth shell surface of the cylindrical filter are not considered in the model. The conductivity of the substrate $\sigma_{\mathrm{bulk}}$ and its fraction $\nu_{\mathrm{bulk}}$ are varied according to Table 1 for the same values as in the resonator setup. In addition, the influence of cell density and, thus, the size of the individual channels will be investigated. This is chosen in such a way that there is always a whole number of channels across the diameter of the cylindrical model. The resonant cavity is completely filled by the filter, which has a diameter of 5 cm and a length of 12.7 cm (5 inch). Such a small diameter was chosen in order to be able to simulate cell densities typical for GPFs, despite the high computational effort. Thus, the largest simulated cell density is 188 cpsi (cells per square inch) and, therefore, only slightly below 200 cpsi of the GPF used for validation measurements in Section 4. The resonant parameters are evaluated for the TE_111_ mode, which has the lowest possible resonant frequency for this geometry and is often used in RF sensor applications.

**Figure 4:**
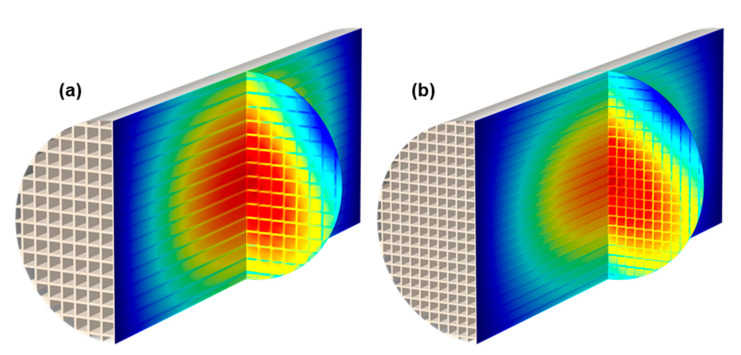
Simulated electric field (red: high field strength; blue: low field strength) of the TE_111_ resonant mode in a monolith filter structure ($\nu_{\mathrm{bulk}}$= 30%; $\sigma_{\mathrm{bulk}}$ = 10 mS/m) with different cell densities: (**a**) 58 cpsi; (**b**) 114 cpsi.

In Figure 4, clear differences in field strength between the substrate and the intervening air can be seen, similar to those in the resonator setup. However, a different polarization behavior related to cell density, as in the case for different numbers of layer in Figure 3, cannot be observed.

The modal analysis of the simulation model provides the resulting resonant frequency f for each simulated parameter combination as well as the inverse quality factor *Q^−^*^1^. Only the latter is relevant for the application of mixing rules concerning the dielectric losses $\varepsilon_{r}^{\prime \prime }$ (cf. Equation (3)). Since they are primarily relevant for signal changes of the RF sensor, in this work, only the quality factor will be evaluated in further detail.

**Figure 5:**
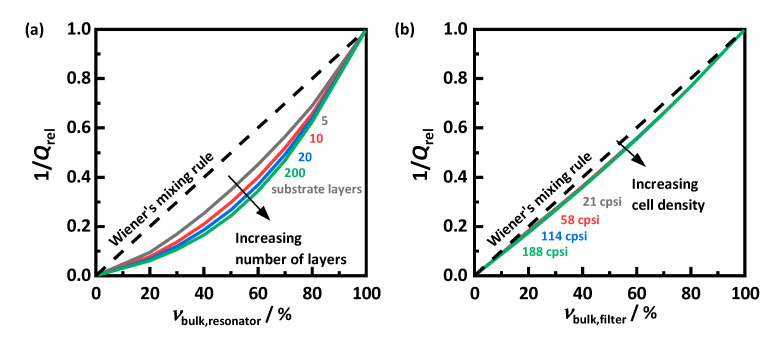
Inverse quality factor $Q^{-1}$ depending on the bulk fraction $\nu_{\mathrm{bulk}}$ at a substrate conductivity $\sigma_{\mathrm{bulk}}$ of 100 mS/m for (**a**) different numbers of substrate layers in the resonator setup, respectively, and (**b**) different cell densities in the filter setup according to Table 1 and as indicated by the color-coded labels within the figure. Dashed line: Wiener’s mixing rule for a series circuit (*k* = 1) in Equation (5).

For an easier assessment of the air content dependency, the inverse quality factor is shown in Figure 5, normalized to the value for the non-porous sample (i.e., $\nu_{\mathrm{bulk}}$ = 1) for each parameter combination. In absence of a substrate, $Q^{-1}$ drops to zero, since no dielectric losses are induced by air. The characteristics of the displayed curves behave according to the related mixing rule. Based on the exponential mixing rule approach (Equation (4)) and the correlation between dielectric losses and quality factor (Equation (3)), the effective quality factor $Q_{\mathrm{eff}}^{-1}$ for mixtures of filter substrate and a lossless mixture component can be described as follows:

(5) $$Q_{\mathrm{eff}}^{-1}=v_{\mathrm{bulk}}{}^{1/k}\cdot Q^{-1}\left(v_{\mathrm{bulk}}=1\right).$$

As expected from the polarization effect apparent in the field images, the effective values in both the resonator and the filter setup deviate from Wiener’s mixing rule (*k* = 1). This effect is much more pronounced for the resonator sample than in the monolith structure. Furthermore, the different strength of polarization resulting from the substrate layer number leads to significantly varying mixing rules. For the filter setup, this influence is almost inexistent.

**Figure 6:**
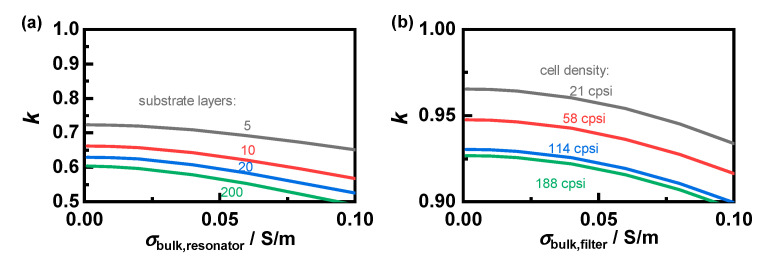
Exponent of the applicable mixing rule k depending on the substrate conductivity $\sigma_{\mathrm{bulk}}$ for (**a**) a different number of substrate layers in the resonator setup, respectively, and (**b**) different cell densities in the filter setup, according to Table 1. Please note: The ordinate axes scaling is enlarged to highlight the differences.

For a more precise evaluation of the power law-based mixing rules, their exponent *k* is determined (Figure 6). For this purpose, a fit curve following Equation (5) is adjusted to the simulated resonant parameters using a nonlinear least-squares approach. This evaluation reveals a significantly stronger deviation from Wiener’s mixing rule for samples in the resonator setup. The exponent *k* decreases slightly with increasing substrate conductivity, resulting in a more pronounced mixing rule and, thus, lower effective losses relative to those of the substrate itself.

For both setups, the mixing rule is affected by the number of simulated layers respective to the cell density. However, with increasing fineness, the exponent *k* approaches a limit. In the resonator setup, doubling from 10 to 20 layers leads to a similar decrease of *k* as the following tenfold increase to 200. Similarly, increasing the cell density of the simulated filter from 114 to 188 cpsi has a much smaller effect than previous changes. Thus, it can be assumed that further increase will not lead to further significant changes of the mixing rules. Therefore, the determined exponent for 200 substrate layers and for a cell density of 188 cpsi is applied for the subsequent measurements. Based on those mixing rules, it is now possible to infer between the properties of soot-loaded filter substrates and the effective properties of arbitrary mixtures with air, which can be measured using the RF sensor.

### 3.2. Soot-Substrate Mixing Rule

**Figure 7:**
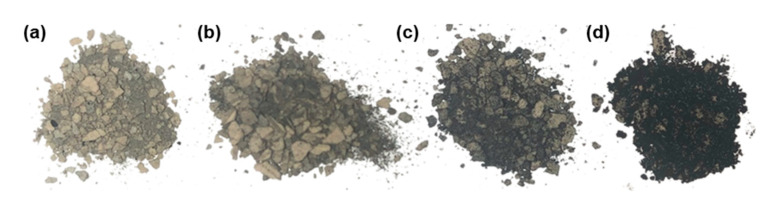
Various PrintexU–cordierite mixtures with a volume fraction $\nu_{\mathrm{soot}}$ of PrintexU of: (**a**) 0.5%, (**b**) 1.5%, (**c**) 5.0%, (**d**) 20%.

In order to derive the soot properties itself, the influence of the filter substrate on the effective losses must be considered. However, determining the mixing rule is therefore not easily feasible by simulation. The size of soot particles is in the ten-nanometer range, while the filter walls, in whose pores the soot is deposited, are several hundred micrometers thick. Furthermore, the soot morphology of individual particles differs widely [51]. A calculation of the field distribution across an entire soot-loaded filter wall, accounting for the shape of every soot particle, as would be necessary to address all effects of mixing rules, does not seem reasonable from a computational point of view. In contrast, this mixing rule can be determined much more easily by measurement. By using synthetic soot, any mixture of filter substrate and soot can be prepared. In this work, PrintexU (Orion Engineered Carbons) is chosen for this purpose, as it is frequently used as artificial soot in automotive applications [52,53]. These investigations could also be carried out with real engine soot. However, only small quantities of engine soot are available, and during soot generation, minor differences, e.g., in engine load or fuel properties, can have an impact on its dielectric properties [8]. Replication of measurements with engine soot would thus be limited due to the uncertain dielectric properties of one of the mixture components. The filter substrate for these investigations is the cordierite from the same GPFs as used for the measurements in Section 4. Some of the measured mixtures of filter substrate and artificial soot are shown in Figure 7.

**Figure 8:**
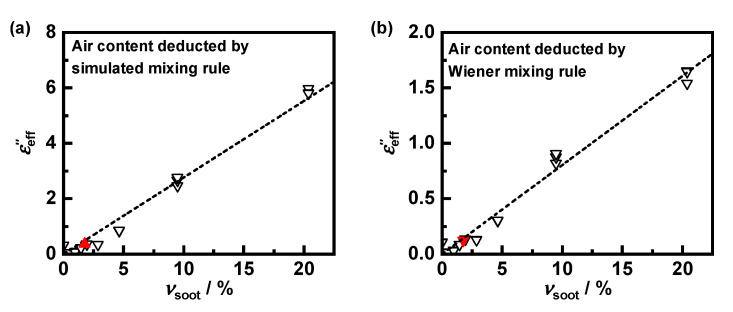
Effective dielectric losses of PrintexU–cordierite mixture $\varepsilon_{\mathrm{eff}}^{\prime \prime }$ depending on the volume fraction of PrintexU $\nu_{\mathrm{soot}}$; the influence of the air fraction was eliminated (**a**) by the mixing rule determined in Section 3.1 and (**b**), for comparison, by Wiener’s mixing rule. The data were measured with the smaller resonator (black triangles) as well as with the larger resonator under consideration of depolarization effects (red triangle); possible linear correlation (i.e., Wiener’s mixing rule) for mixtures between substrate and soot illustrated by the dotted regression line.

Measurements on samples with high soot content or even on pure soot were not possible using the MCP method. Due to its high conductivity, the resonant modes are attenuated severely, preventing evaluation of their parameters. Even with a reduced sample volume, an exact determination would no longer be possible due to strong depolarization effects. Thus, only samples up to a maximum PrintexU content $\nu_{\mathrm{soot}}$ of 20% could be measured. Due to the differences in density between cordierite (2.5 g/cm³) and PrintexU (1.0 g/cm³), this corresponds to a mass fraction of less than 10%. Nevertheless, soot loadings higher than this are not relevant for GPF applications. The mixing behavior has therefore only to be known for small soot contents, such as those investigated in this work. To avoid uncertainties caused by the correction of depolarization effects, the effective dielectric losses were measured using the smaller resonator. The influence of the air content of around 80% was deduced from the obtained values using the previously determined mixing rule (Figure 8a). To estimate its effect on the soot–substrate mixing rule, the effective parameters were also calculated using Wiener’s mixing rule (Figure 8b). Although the application of different mixing rules results in different absolute values of $\varepsilon_{\mathrm{eff}}^{\prime \prime }$, their linear relation to the soot content remains almost unchanged.

As reference, a sample with a soot content of 1.7%, which corresponds to a soot loading of the intact filter of 2 g_soot_/L_GPF_, was measured in the larger resonator. The hereby measured dielectric losses agree well with those from the smaller resonator for the same amount of soot. The effective dielectric losses of PrintexU-loaded cordierite substrate $\varepsilon_{\mathrm{eff}}^{\prime \prime }$, show an almost linear relationship with the soot volume fraction $\nu_{\mathrm{soot}}$. Thus, Wiener’s mixing rule can be applied for mixtures of soot and filter substrate. Along with the simulation-based determined mixing rule to account the air content, it is now possible to deduce the dielectric losses of soot from effective sample properties.

## 4. Validation of Mixing Rules by Engine Test Bench Measurements

**Figure 9:**
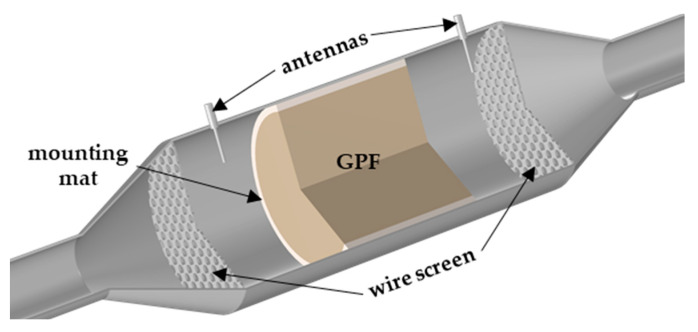
Geometry of the simulation model; the grids before and after the GPF were not considered in the flow simulation.

To verify the accuracy of the determined mixing rules, the RF signal during GPF monitoring on an engine test bench is compared with data obtained by the simulation model presented in [10], in which the effective dielectric properties are calculated using both mixing rules. The engine test bench consists of a 1.8 L direct injection gasoline engine. An uncoated cordierite GPF (5 inch length; 5.2 inch diameter; 200 cpsi cell density; 8.5 mil wall thickness) is installed downstream of a close-coupled three-way catalyst. To monitor the filter using the RF sensor, coaxial antennas are mounted up- and downstream of the filter. To define the resonant cavity more precisely, wire screens are inserted before and after the GPF parallel to the filter front. The coupling elements disturb the electrical field distribution only marginally; nevertheless, it was considered in the simulation model. The power transmitted between the antennas is measured via the scattering parameter *S*_21_ using the vector network analyzer (VNA) Anritsu MS2025B. Due to the low exciting electrical fields, a linear system regarding the interaction with the dielectric properties can be assumed and was used in the simulations, as in the determination of the mixing rules before. This setup, as constructed in the simulation model, can be seen in Figure 9.

**Figure 10:**
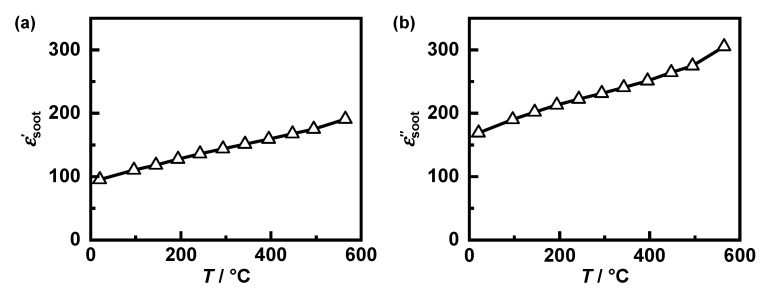
Dielectric properties ((**a**) permittivity; (**b**) dielectric losses) of soot generated on the engine test bench at load corresponding to a velocity at 160 km/h depending on its temperature.

For the experiments, the engine was operated at a steady load point corresponding to a driving speed of 160 km/h. To achieve higher particulate emissions and, therefore, faster filter loading, the engine was operated under rich conditions, contrary to the standard settings of the engine control unit. To measure the dielectric properties of the generated soot, a filter loaded under these conditions was demounted, coarsely crushed and measured in the larger resonator shown in Figure 1a over a temperature range from 20 to 600 °C. In order to derive the soot properties as accurately as possible, the dielectric properties of cordierite have to be known to consider them for the mixing rule of the components soot and substrate. For this purpose, the soot was removed from the sample still present in the resonator with an oxidizing gas atmosphere at 600 °C. Subsequently, the same sample could be measured again to obtain the soot-free substrate properties. Using the MCP method described in [27] as well as the mixing rules from Section 3, the dielectric properties of the generated soot were be determined (Figure 10). The mixing rules were also applied besides the losses $\varepsilon_{\mathrm{soot}}^{\prime \prime }$ for the permittivity $\varepsilon_{\mathrm{soot}}^{\prime }$. Both parameters increase significantly with temperature, although, regardless of this, the losses are slightly higher than the permittivity.

The now-known soot properties are included in the simulation model. Depending on the calculated filter temperature and the present soot loading, the effective GPF properties are calculated via mixing rules. Based on this, the simulation model can calculate the theoretical signal of the RF sensor at arbitrary engine conditions. To validate whether the simulation model and, consequently, the determined mixing rules can calculate the RF signal correctly, its behavior during filter loading, interrupted by several partial regenerations, is simulated using input data from an engine test bench measurement and then compared with the real sensor signal. Such partial regenerations are examined as they occur frequently in gasoline applications and can lead to a reduced accurate soot monitoring by differential pressure sensors [9,13,16]. For instance, after several partial regenerations, a lower differential pressure than during initial loading can be measured, while a simultaneously operated RF sensor shows no hysteresis [17].

**Figure 11:**
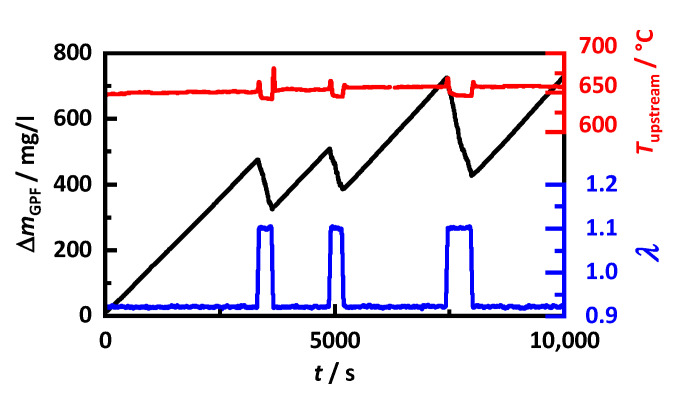
Exhaust gas data during soot loading with multiple partial regenerations caused by switching to lean operation; amount of soot accumulated in the GPF ${\Delta m}_{\mathrm{GPF}}$ (black), temperature $T_{\mathrm{upstream}}$ (red) and air-fuel ratio λ (blue) upstream of the GPF.

During the experiments, the engine was operated as before at a load corresponding to 160 km/h driving speed. The partial regenerations were initiated by switching the air-fuel ratio from rich to lean. This results in a slight decrease in the exhaust gas temperature (Figure 11). The amount of soot accumulated in the GPF ${\Delta m}_{\mathrm{GPF}}$, is calculated by integration via the soot concentration in the exhaust gas measured by an AVL 483 MicroSoot sensor. In addition, the amount of oxidized soot during regeneration is considered using lambda probes up- and downstream of the filter, measuring the oxygen consumption during soot regeneration.

**Figure 12:**
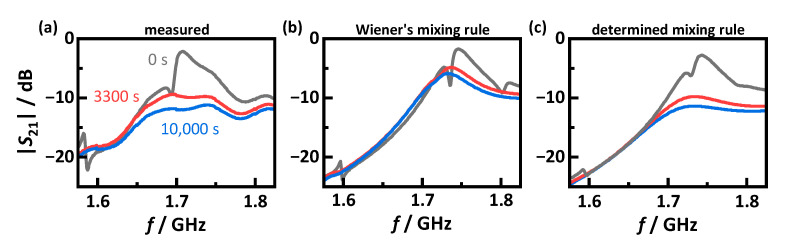
Frequency spectra of the transmission parameter $\left|S_{21}\right|$ at different times during filter loading: soot free filter at *t* = 0 s (black), before first regeneration at *t* = 3300 s and 472 mg/L_GPF_ stored soot (red) and after three regenerations and subsequent soot loading at *t* = 10,000 s and 730 mg/L_GPF_ stored soot (blue); (**a**) measured data; (**b**) simulated data using Wiener’s mixing rule to consider the air content; (**c**) simulated data using the mixing rule determined in Section 3.1.

In contrast to the MCP resonator setup, in these measurements, the RF sensor does not evaluate a specific resonant mode. Due to the accumulation of highly conductive soot in the filter, resonant modes are attenuated to such an extent that an analysis of these is no longer possible. To monitor the GPF loading nevertheless, an averaged transmission parameter $\left|S_{21}\right|$ can be evaluated [8,25]. In this work, a frequency range from 1.68 to 1.78 GHz is examined. Therefore, in contrast to the previous modal analysis, the simulation model calculates $\left|S_{21}\right|$ itself, allowing a direct comparison with measured RF-spectra. Figure 12 shows spectra at three different moments: first for the unloaded GPF, second at a soot load of 472 mg/L_GPF_ before the first regeneration and third at 730 mg/L_GPF_ after three partial regenerations and a subsequent soot loading. The spectra simulated with the mixing rules determined in Section 3 (Figure 12c) agree well with the measured spectra both for the unloaded and for the two loaded state. For comparison, the simulations were also performed with deviating mixing rules. Thus, for the spectra shown in Figure 12b, the air content of the sample respective to the filter was considered using Wiener’s rule instead of the rule determined in Section 3.1.

For the soot-free GPF, the spectra agree well regardless of the applied mixing rule. This could be due the low losses of the substrate. Although the effective losses $\varepsilon_{\mathrm{eff}}^{\prime \prime }$ averaged over the GPF using the determined mixing rules are more than twice as high compared to Wiener’s mixing rule, those losses are negligible relative to the conductivity increase due to the deposited soot. Thus, $\varepsilon_{\mathrm{eff}}^{\prime \prime }$ is increased by a factor of more than ten by the end of the measurement. Nevertheless, smaller differences between the simulated spectra exist, especially at antiresonances as they occur, e.g., at 1.8 GHz, which are more pronounced with applying Wiener’s rule. This is different for spectra in the soot-loaded state. Those computed on the basis of Wiener’s rule show a considerably underestimated signal attenuation. With the mixing rules from Section 3, on the other hand, the simulations agree very well with the measurements, even for soot-loaded GPFs.

**Figure 13:**
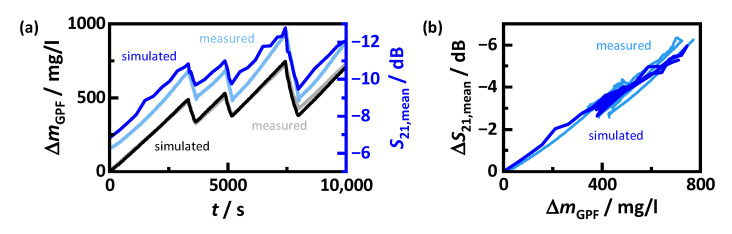
(**a**) Measured (see text to Figure 11) and simulated amount of soot stored in the GPF ${\Delta m}_{\mathrm{GPF}}$ as well as RF signal attenuation $S_{21,\mathrm{mean}}$ (averaged in the frequency range from 1.68 to 1.78 GHz) during filter loading; (**b**) measured and simulated change in the mean RF signal attenuation ${\Delta S}_{21,\mathrm{mean}}$ depending on the stored soot ${\Delta m}_{\mathrm{GPF}}$.

In order to validate the predictions of the simulation model in more detail, Figure 13a shows the soot loading ${\Delta m}_{\mathrm{GPF}}$ as well as the averaged transmission parameter $S_{21,\mathrm{mean}}$ for measurement and simulation. The amount of stored soot can be predicted very accurately. At the end of the measurement, the model predicts a loading that is only 20 mg/L_GPF_ too low. The RF signal, on the other hand, deviates more and predicts a slightly higher attenuation. This may be not only due to a slightly incorrect prediction of the filter permittivity, but also caused by modeling antennas and canning not fully in detail.

More relevant for evaluating the accuracy of the RF simulation with respect to soot load prediction is the change of the transmission signal ${\Delta S}_{21,\mathrm{mean}}$, as it represents the changing dielectric losses due to soot deposition (Figure 13b). Its dependency with the soot loading agrees almost perfectly with the measured values. The average deviation in these investigations between simulation and measurement is only 0.2%. Individual data points may deviate more, but as with the absolute transmission signal, this may be a consequence of the imperfect simulation geometry.

Overall, the validation measurements show that the mixing rules established in this work allow the dielectric properties of gasoline soot to be determined with such accuracy that a simulation model can replicate the RF sensor behavior with high precision.

## 5. Conclusions

In this study, mixing rules were investigated and developed to deduce the dielectric properties of soot originating from a whole particulate filter. These rules, along with the procedure described in [27], allow crushed filter samples to be measured using the microwave cavity perturbation (MCP) method. Therefore, two mixing rules have to be considered. One describes the interaction between filter substrate and soot deposited on it. Measurements on a resonator setup have shown that Wiener’s mixing rule is applicable for this. The other mixing rule then describes the interaction between the soot-loaded substrate and the surrounding air. By simulation, a mixing rule following a power approach could be found. The resulting exponent depends strongly on the observed geometry. Thus, the applicable mixing rule for an intact monolithic filter structure deviates less from Wiener’s rule than for a crushed filter in the resonator setup. The accuracy of the mixing rules was verified by comparative measurements of the RF sensor on an engine test bench. For this purpose, engine soot was measured by the MCP method using the determined mixing rules. The resulting dielectric properties were included in the simulation model described in [10], which allowed the real engine test bench measurement to be re-simulated. A comparison with measured data showed high agreement of the measured RF signal, which would not be the case with deviating mixing rules. Thus, the mixing rules determined in this work allow a more accurate determination of the dielectric properties of gasoline soot on GPFs. This allows a more accurate simulation-based prediction of the RF sensor behavior, supporting the sensor development for GPF applications.

## Figures and Tables

**Table 1:** Parameter variation in the simulation models. Besides the bulk volume fraction of the sample $\nu_{\mathrm{bulk}}$ and also its conductivity $\sigma_{\mathrm{bulk}}$, (a) in case of the resonator model, the layer number and (b) for the filter structure, the cell density was varied (cpsi stands for cells per square inch).

$\nu_{\mathrm{bulk}}$ **/%**	0/20/30/40/50/60/70/80/100
$\sigma_{\mathrm{bulk}}$ **/mS/m**	1/10/20/40/60/80/100
**(a) substrate layers**	5/10/20/200
**(b) cell density/cpsi**	21/58/114/188

## Data Availability

All relevant data presented in the article are stored according to institutional requirements and as such are not available online. However, all data used in this paper can be made available upon request to the authors.

## References

[1] Hennig F., Quass U., Hellack B., Küpper M., Kuhlbusch T.A.J., Stafoggia M., Hoffmann B. (2018). Ultrafine and Fine Particle Number and Surface Area Concentrations and Daily Cause-Specific Mortality in the Ruhr Area, Germany, 2009–2014. Environ. Health Perspect..

[2] Soppa V.J., Shinnawi S., Hennig F., Sasse B., Hellack B., Kaminski H., Quass U., Schins R.P.F., Kuhlbusch T.A.J., Hoffmann B. (2019). Effects of short-term exposure to fine and ultrafine particles from indoor sources on arterial stiffness—A randomized sham-controlled exposure study. Int. J. Hyg. Environ. Health.

[3] Kittelson D.B. (1998). Engines and nanoparticles. J. Aerosol Sci..

[4] Chen L., Liang Z., Zhang X., Shuai S. (2017). Characterizing particulate matter emissions from GDI and PFI vehicles under transient and cold start conditions. Fuel.

[5] Schoenhaber J., Kuehn N., Bradler B., Richter J.M., Bauer S., Lenzen B., Beidl C. (2017). Impact of European Real-Driving-Emissions Legislation on Exhaust Gas Aftertreatment Systems of Turbocharged Direct Injected Gasoline Vehicles. SAE Tech. Pap..

[6] Demuynck J., Favre C., Bosteels D., Hamje H., Andersson J. (2017). Real-World Emissions Measurements of a Gasoline Direct Injection Vehicle without and with a Gasoline Particulate Filter. SAE Tech. Pap..

[7] Rose D., Boger T. (2009). Different Approaches to Soot Estimation as Key Requirement for DPF Applications. SAE Tech. Pap..

[8] Walter S., Schwanzer P., Hagen G., Haft G., Dietrich M., Rabl H.-P., Moos R., Tille T. (2020). Hochfrequenzsensorik zur direkten Beladungserkennung von Benzinpartikelfiltern. Automobil-Sensorik 3.

[9] Gaiser G., Mucha P. (2004). Prediction of Pressure Drop in Diesel Particulate Filters Considering Ash Deposit and Partial Regenerations. SAE Tech. Pap..

[10] Walter S., Schwanzer P., Hagen G., Haft G., Rabl H.-P., Dietrich M., Moos R. (2020). Modelling the Influence of Different Soot Types on the Radio-Frequency-Based Load Detection of Gasoline Particulate Filters. Sensors.

[11] Liu X., Chanko T., Lambert C., Maricq M. (2018). Gasoline Particulate Filter Efficiency and Backpressure at Very Low Mileage. SAE Tech. Pap..

[12] Lambert C., Chanko T., Dobson D., Liu X., Pakko J. (2017). Gasoline Particle Filter Development. Emiss. Control Sci. Technol..

[13] Saito C., Nakatani T., Miyairi Y., Yuuki K., Makino M., Kurachi H., Heuss W., Kuki T., Furuta Y., Kattouah P. (2011). New Particulate Filter Concept to Reduce Particle Number Emissions. SAE Tech. Pap..

[14] Chan T.W., Meloche E., Kubsh J., Rosenblatt D., Brezny R., Rideout G. (2012). Evaluation of a Gasoline Particulate Filter to Reduce Particle Emissions from a Gasoline Direct Injection Vehicle. SAE Int. J. Fuels Lubr..

[15] Wang-Hansen C., Ericsson P., Lundberg B., Skoglundh M., Carlsson P.-A., Andersson B. (2013). Characterization of Particulate Matter from Direct Injected Gasoline Engines. Top. Catal..

[16] Suresh A., Khan A., Johnson J.H. (2000). An Experimental and Modeling Study of Cordierite Traps—Pressure Drop and Permeability of Clean and Particulate Loaded Traps. SAE Tech. Pap..

[17] Schwanzer P., Mieslinger J., Dietrich M., Haft G., Walter S., Hagen G., Moos R., Gaderer M., Rabl H.-P. Monitoring of a Particulate Filter for Gasoline Direct Injection Engines with a Radio-Frequency-Sensor. Proceedings of the 11th Internationales Symposium für Abgasund Partikelemissionen.

[18] Sethia S., Kubinski D., Nerlich H., Naber J. (2020). RF Studies of Soot and Ammonia Loadings on a Combined Particulate Filter and SCR Catalyst. J. Electrochem. Soc..

[19] Moos R. (2015). Microwave-Based Catalyst State Diagnosis—State of the Art and Future Perspectives. SAE Int. J. Engines.

[20] Sappok A., Bromberg L., Parks J.E., Prikhodko V. (2010). Loading and Regeneration Analysis of a Diesel Particulate Filter with a Radio Frequency-Based Sensor. SAE Tech. Pap..

[21] Sappok A., Bromberg L. (2014). Radio Frequency Diesel Particulate Filter Soot and Ash Level Sensors: Enabling Adaptive Controls for Heavy-Duty Diesel Applications. SAE Int. J. Commer. Veh..

[22] Feulner M., Hagen G., Hottner K., Redel S., Müller A., Moos R. (2017). Comparative Study of Different Methods for Soot Sensing and Filter Monitoring in Diesel Exhausts. Sensors.

[23] Feulner M., Hagen G., Piontkowski A., Müller A., Fischerauer G., Brüggemann D., Moos R. (2013). In-Operation Monitoring of the Soot Load of Diesel Particulate Filters: Initial Tests. Top. Catal..

[24] Nicolin P., Boger T., Dietrich M., Haft G., Bachurina A. (2020). Soot Load Monitoring in Gasoline Particulate Filter Applications with RF-Sensors. SAE Tech. Pap..

[25] Dietrich M., Jahn C., Lanzerath P., Moos R. (2015). Microwave-Based Oxidation State and Soot Loading Determination on Gasoline Particulate Filters with Three-Way Catalyst Coating for Homogenously Operated Gasoline Engines. Sensors.

[26] Beulertz G., Fritsch M., Fischerauer G., Herbst F., Gieshoff J., Votsmeier M., Hagen G., Moos R. (2013). Microwave Cavity Perturbation as a Tool for Laboratory In Situ Measurement of the Oxidation State of Three Way Catalysts. Top. Catal..

[27] Steiner C., Walter S., Malashchuk V., Hagen G., Kogut I., Fritze H., Moos R. (2020). Determination of the Dielectric Properties of Storage Materials for Exhaust Gas Aftertreatment Using the Microwave Cavity Perturbation Method. Sensors.

[28] Dietrich M., Rauch D., Porch A., Moos R. (2014). A Laboratory Test Setup for in Situ Measurements of the Dielectric Properties of Catalyst Powder Samples under Reaction Conditions by Microwave Cavity Perturbation: Set up and Initial Tests. Sensors.

[29] Sihvola A. (2000). Mixing Rules with Complex Dielectric Coefficients. Subsurf. Sens. Technol. Appl..

[30] Pozar D.M. (2012). Microwave Engineering.

[31] Chen L. (2005). Microwave Electronics: Measurement and Materials Characterization.

[32] Parkash A., Vaid J.K., Mansingh A. (1979). Measurement of Dielectric Parameters at Microwave Frequencies by Cavity-Perturbation Technique. IEEE Trans. Microw. Theory Tech..

[33] Venermo J., Sihvola A. (2005). Dielectric polarizability of circular cylinder. J. Electrost..

[34] Jylhä L., Sihvola A. (2007). Equation for the effective permittivity of particle-filled composites for material design applications. J. Phys. D Appl. Phys..

[35] Cheng E.M., Malek M.F.b.A., Ahmed M., You K.Y., Lee K.Y., Nornikman H. (2012). The Use of Dielectric Mixture Equations to Analyze the Dielectric Properties of a Mixture of Rubber Tire Dust and Rice Husks in a Microwave Absorber. Prog. Electromagn. Res..

[36] Marquardt P. (1987). Quantum-size affected conductivity of mesoscopic metal particles. Phys. Lett. A.

[37] El Bouazzaoui S., Achour M.E., Brosseau C. (2011). Microwave effective permittivity of carbon black filled polymers: Comparison of mixing law and effective medium equation predictions. J. Appl. Phys..

[38] Jusoh M., Abbas Z., Hassan J., Azmi B., Ahmad A. (2011). A Simple Procedure to Determine Complex Permittivity of Moist Materials Using Standard Commercial Coaxial Sensor. Meas. Sci. Rev..

[39] Camacho Hernandez J.N., Link G., Soldatov S., Füssel A., Schubert M., Hampel U. (2020). Experimental and numerical analysis of the complex permittivity of open-cell ceramic foams. Ceram. Int..

[40] Camerucci M.A., Urretavizcaya G., Castro M.S., Cavalieri A.L. (2001). Electrical properties and thermal expansion of cordierite and cordierite-mullite materials. J. Eur. Ceram. Soc..

[41] Nelson S.O. (2005). Density-Permittivity Relationships for Powdered and Granular Materials. IEEE Trans. Instrum. Meas..

[42] Tuhkala M., Juuti J., Jantunen H. (2014). An indirectly coupled open-ended resonator applied to characterize dielectric properties of MgTiO_3_–CaTiO_3_ powders. J. Appl. Phys..

[43] Dube D.C. (1970). Study of Landau-Lifshitz-Looyenga’s formula for dielectric correlation between powder and bulk. J. Phys. D Appl. Phys..

[44] Looyenga H. (1965). Dielectric constants of heterogeneous mixtures. Physica.

[45] Birchak J.R., Gardner C.G., Hipp J.E., Victor J.M. (1974). High dielectric constant microwave probes for sensing soil moisture. Proc. IEEE.

[46] Goncharenko A.V. (2003). Generalizations of the Bruggeman equation and a concept of shape-distributed particle composites. Phys. Review. E Stat. Nonlinear Soft Matter Phys..

[47] Maxwell Garnett J.C. (1906). Colours in Metal Glasses, in Metallic Films, and in Metallic Solutions. II. Philos. Trans. R. Soc. Lond. Ser. A Contain. Pap. A Math. Phys. Character.

[48] Lichtenecker K., Rother K. (1931). Die Herleitung des logarithmischen Mischungsgesetzes aus allgemeinen Prinzipien der stationären Strömung. Phys. Zeitschr..

[49] Wiener O. (1910). Zur Theorie der Refraktionskonstanten. Math. Phys. Kl..

[50] Karkkainen K.K., Sihvola A.H., Nikoskinen K.I. (2000). Effective permittivity of mixtures: Numerical validation by the FDTD method. IEEE Trans. Geosci. Remote Sens..

[51] Seong H., Choi S., Lee K. (2014). Examination of nanoparticles from gasoline direct-injection (GDI) engines using transmission electron microscopy (TEM). Int. J. Automot. Technol..

[52] Boger T., Rose D., Nicolin P., Gunasekaran N., Glasson T. (2015). Oxidation of Soot (Printex^®^ U) in Particulate Filters Operated on Gasoline Engines. Emiss. Control Sci. Technol..

[53] Choi S., Seong H. (2015). Oxidation characteristics of gasoline direct-injection (GDI) engine soot: Catalytic effects of ash and modified kinetic correlation. Combust. Flame.

